# Members of the Chloride Intracellular Ion Channel Protein Family Demonstrate Glutaredoxin-Like Enzymatic Activity

**DOI:** 10.1371/journal.pone.0115699

**Published:** 2015-01-12

**Authors:** Heba Al Khamici, Louise J. Brown, Khondker R. Hossain, Amanda L. Hudson, Alxcia A. Sinclair-Burton, Jane Phui Mun Ng, Elizabeth L. Daniel, Joanna E. Hare, Bruce A. Cornell, Paul M. G. Curmi, Mary W. Davey, Stella M. Valenzuela

**Affiliations:** 1 School of Medical and Molecular Biosciences, University of Technology Sydney, Sydney, New South Wales 2007, Australia; 2 Centre for Health Technologies, University of Technology Sydney, Sydney, New South Wales 2007, Australia; 3 Department of Chemistry and Bimolecular Sciences, Macquarie University, Sydney, New South Wales 2109, Australia; 4 Surgical Diagnostics, Roseville, Sydney, New South Wales 2069, Australia; 5 School of Physics, University of New South Wales, Sydney, New South Wales 2052, Australia; 6 Centre for Applied Medical Research, St Vincent's Hospital, Sydney, New South Wales 2010, Australia; 7 Bragg Institute, Australian Nuclear Science and Technology Organisation, Sydney, New South Wales 2234, Australia; Instituto de Biociencias - Universidade de São Paulo, Brazil

## Abstract

The Chloride Intracellular Ion Channel (CLIC) family consists of six evolutionarily conserved proteins in humans. Members of this family are unusual, existing as both monomeric soluble proteins and as integral membrane proteins where they function as chloride selective ion channels, however no function has previously been assigned to their soluble form. Structural studies have shown that in the soluble form, CLIC proteins adopt a glutathione S-transferase (GST) fold, however, they have an active site with a conserved glutaredoxin monothiol motif, similar to the omega class GSTs. We demonstrate that CLIC proteins have glutaredoxin-like glutathione-dependent oxidoreductase enzymatic activity. CLICs 1, 2 and 4 demonstrate typical glutaredoxin-like activity using 2-hydroxyethyl disulfide as a substrate. Mutagenesis experiments identify cysteine 24 as the catalytic cysteine residue in CLIC1, which is consistent with its structure. CLIC1 was shown to reduce sodium selenite and dehydroascorbate in a glutathione-dependent manner. Previous electrophysiological studies have shown that the drugs IAA-94 and A9C specifically block CLIC channel activity. These same compounds inhibit CLIC1 oxidoreductase activity. This work for the first time assigns a functional activity to the soluble form of the CLIC proteins. Our results demonstrate that the soluble form of the CLIC proteins has an enzymatic activity that is distinct from the channel activity of their integral membrane form. This CLIC enzymatic activity may be important for protecting the intracellular environment against oxidation. It is also likely that this enzymatic activity regulates the CLIC ion channel function.

## Introduction

The Chloride Intracellular Channel (CLIC) proteins are highly conserved in vertebrates, with the following six members found in humans: CLIC1 [1], CLIC2 [2], CLIC3 [3], CLIC4 [4], [5], CLIC5 [6] and CLIC6 [7]. The CLICs exist as both globular soluble and as integral membrane proteins. These proteins are known to spontaneously transit from their soluble state into an integral membrane form, where they can act as anion selective channels [4], [8]–[10]. CLIC1 channel conductance is regulated by a number of factors including cholesterol [11], redox [12], [13] membrane phospholipid composition and pH [9], [10].

Determining the cellular function of the CLICs in vertebrates has proven difficult due to the presence of six members, suspected of functional redundancy in knock-out model systems [14]. To date, knock-out mouse models have been established for CLIC1 [15], [16] CLIC4 [17], [18] and CLIC5 [19] with each demonstrating distinct phenotypes. From such studies, it is postulated that individual CLIC protein members are involved in regulation of processes including cell growth, cell division and apoptosis [18]–[22] acidification of intracellular organelles [23], [24], formation of stereocilia [19] and development of the organ of Corti [25], [26].

Structural studies have shown that in their soluble form the CLIC proteins are members of the glutathione S-transferase (GST) fold family of proteins [8], [27]. The GSTs can be divided into at least twelve classes of multifunctional enzymes that exist largely as dimeric proteins in the cytosolic environment of cells [28]. They are well known for their ability to catalyse the conjugation of glutathione (GSH) to exogenous toxins and xenobiotics, and therefore vital in the detoxification processes within cells [29]. They are also involved in the synthesis of prostaglandins [30], and facilitate the intracellular transport of hydrophobic compounds [30]. GSTs are reported to have additional functions including the binding of bilirubin and carcinogens, and their over-expression in tumour cells was found to contribute to anticancer drug resistance [29], [31], [32].

The GST- omega class proteins, as distinct to other GSTs, exhibit glutathione-dependent thiol transferase activity and have been shown to catalyse the glutathione-dependent reduction of dehydroascorbate (DHA) [33], [34]. The enzymatic activity of the GST-omega proteins resembles that of the glutaredoxins [33], which are structurally related to the thioredoxins and are involved in the reduction of intracellular disulfides by catalysing reactions that couple GSH, NADPH and glutathione reductase (GR), contributing to the maintenance of a healthy redox environment within cells [29], [35], [36]. Like the GSTs, members of the glutaredoxin family contain a GSH binding site within their conserved thioredoxin domain known as the G-site [33]. The glutaredoxin G-site is either monothiol, containing a single cysteine residue [**Cys**-Gly-Phe-Ser] or dithiol [**Cys**-Pro-Tyr-**Cys**]. Protein members in the latter group generally act as thiol-disulfide oxidoreductases (via a dithiol mechanism), while the monothiol members act as detoxifying or stress response proteins, by forming mixed disulfides between GSH and target proteins, or low-molecular weight thiols [37].

**Figure 1:**
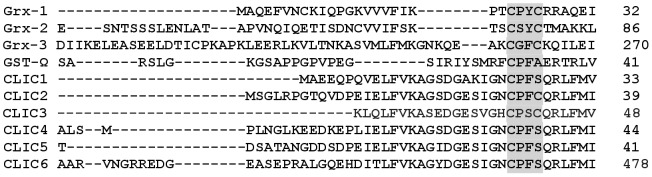
Conserved G-site motif in members of the CLIC family. Multiple sequence alignment of human proteins: CLIC 1-6, GST-omega and Grx1-3. Highlighted in grey is the glutaredoxin/thioredoxin active site motif (G-site) (Accession numbers: CLIC1 (CAG46868), CLIC2 (CAG03948), CLIC3 (CAG46863.1), CLIC4 (CAG38532), CLIC5 (AAF66928), CLIC6 (NP_444507), GST-omega (AAF73376), Grx-1 (BAAO4769), Grx-2 (AAK83089) and Grx-3 (AAH0528289) obtained from ClustalW.

X-ray crystallography revealed that the soluble form of CLIC1 adopts a three dimensional fold similar to the GST superfamily, and in particular the GST-omega class [8], [27]. The CLIC1 structure consists of an all alpha-helical C-terminal domain and an N-terminal thioredoxin domain comprised of four beta-strands sandwiched between three alpha-helices that contains the glutaredoxin-like monothiol motif [**Cys**-Pro-Phe-Ser]. The active cysteine residue, Cys24 in CLIC1, was found to covalently bind GSH in a manner similar to the GST-omega proteins that possess a monothiol G-site [**Cys**-Pro-Phe-Ala] [33]. Interestingly, CLICs 2 and 3 [8] contain the dithiol motif [**Cys**-X-X-**Cys**], while CLICs 1, 4, 5 and 6 contain the monothiol active site motif [**Cys**-X-X-Ser] as shown in Fig. 1.

Due to the high level of structural homology between the CLIC proteins and these well-known redox enzymes [33], it has been speculated that the soluble form of the CLICs would also function as oxidoreductase enzymes [8], [14]. However experimental evidence in support of this hypothesis has until now, not been forthcoming. Our current study demonstrates for the first time, that members of the CLIC protein family exhibit glutaredoxin-like enzymatic activity. Furthermore, our findings provide evidence of a functional activity for the soluble form of these proteins, which appears to be autonomous to their well-characterised membrane ion channel activity, potentially classing them as moonlighting proteins [38]. Finally, we observe that small molecules known to inhibit the CLIC1 ion channel also inhibit its enzymatic activity. This suggests that the enzymatic function of the CLIC proteins may regulate their ion channel activity.

## Materials and Methods

### Materials

The following reagents were all purchased from *Sigma Aldrich*: Recombinant thioredoxin-1 (Trx-1) and glutaredoxin-1 (Grx-1) Thioredoxin reductase (TrxR) from rat liver and glutathione reductase (GR) from yeast, reduced glutathione (GSH), sodium selenite (Na_2_SeO_3_), reduced nicotinamide adenine dinucleotide phosphate (NADPH), dehydroascorbic acid (DHA), 2-hydroxyethyl disulphide (HEDS), cholesterol (99% purity), dithiothreitol (DTT), indanyloxyacetic acid (IAA-94), anthracene-9-carboxylic acid (A9C), and 4,4′-diisothiocyano-2,2′stilbene-disulfonic acid (DIDS), bovine plasma thrombin.

Glutathione Sepharose 4B resin was purchased from GE Healthcare (*Piscataway, USA*). Saxitoxin was supplied by National Research Council (NRC) of Canada Institute of Marine Biosciences (*Halifax, NS, Canada*).

### Expression and Purification of wild-type recombinant CLIC1, CLIC2, CLIC4, HcTrx-5 and CLIC1 mutant proteins

Wild-type CLIC1 protein was expressed in *E. coli* BL21 (DE3) using the His-tag pET28a vector (*Novagen*) as previously described [39]. Briefly, the transformed cells were grown and incubated in 2xYT media at 37°C overnight. The cells were then induced with 1 mM IPTG and allowed to grow for a further 16 hours at 20°C. Soluble fractions of cell lysates were run over Ni^2+^-NTA resin chromatography column. The bound CLIC proteins were cleaved from their His-tag and eluted from the column following incubation with 30 NIH units of bovine plasma thrombin per litre of cell culture for ∼16 hours. 1 mM DTT was added and CLIC1 protein further purified on a Superdex-75 high performance Size Exclusion Chromatography (SEC) column at 4°C. The CLIC1 monomer fraction was eluted and stored in 100 mM KCl, 1 mM NaN_3_, 20 mM HEPES pH 7.5), containing 1 mM DTT in order to maintain the CLIC1 protein in its reduced monomeric form.

CLIC2 in the pGEX-2T vector was expressed in *E. coli* BL21(DE3). This vector coded for an N-terminal GST purification tag from which the full length CLIC2 product was then cleaved via an internal thrombin digestion site. Cells were grown in 2xYT media at 37°C and induced at mid-log phase with 1 mM IPTG before harvesting after an additional 16 hours of growth at 15°C. Soluble CLIC2 was purified on glutathione sepharose 4B resin *(Amersham Bioscience, USA)* and eluted from the column in phosphate buffered saline after overnight incubation with bovine plasma thrombin (∼1 NIH unit/mg). 0.3 mM DTT was then added and the CLIC2 sample further purified on a Superdex 75 SEC column equilibrated in 100 mM KCl, 1 mM NaN_3_, 20 mM HEPES, pH 7.5.

CLIC4 protein was purified as previously described [40]. HcTrx-5 protein from *Haemonchus contortus* was also purified as previously described [41]. Mutant versions of CLIC1-C24A and CLIC1-C59A in pET28a vector were made using the site-directed mutagenesis kit (*Stratagene, USA*) and purified as previously described [39]. The mutant CLIC1-C24S in pGEX-4T-1 *(AMRAD-Pharmacia)* vector was made and purified as previously described [13], [39].

### Purification of recombinant CLIC1 dimer

CLIC1 dimer was prepared as previously described [40]. Briefly, CLIC1 was oxidised by the addition of H_2_O_2_ to a final concentration of 2mM in phosphate buffered saline (pH7.4). The protein was incubated under oxidizing conditions for 2 hours, after which the dimer form was isolated via size exclusion chromatography as outlined previously [42].

### Enzyme assays

Assays were performed in 96-well plates, with a final volume of 200 uL and absorbance read using a BioTek microplate spectrophotometer. All kinetic analyses were performed using Microsoft Excel 2010 and GraphPad Prism 6.

### HEDS Enzyme Assay

Assays were carried out following the method described in [35]. Briefly, either reduced monomeric CLIC1 (WT), oxidised dimeric CLIC1 (WT), CLIC1-C24A, CLIC1-C24S, CLIC1-C59A, CLIC2, CLIC4, HcTrx-5 or Grx-1 (5 uM final concentration) was added to 5 mM potassium phosphate buffer (pH 7) containing 1 mM EDTA, 250 uM NADPH, 50 nM GR and 1 mM HEDS. The mixture was incubated for 5 minutes at 37°C, with the reaction initiated by addition of 1 mM GSH. Consumption of NADPH was monitored at A_340 nm_.

### HEDS Enzyme Assay for CLIC Proteins in the Presence of Thioredoxin Reductase

5 uM final concentration of either CLIC1, CLIC2, CLIC4 or Trx-1 were added to 0.1 M Tris-HCl buffer (pH 7.5) containing 1 mM EDTA, 200 uM NADPH and 50 nM TrxR (from rat liver). The mixture was incubated for 5 minutes at 37°C, with the reaction initiated by addition of 750 uM HEDS. Consumption of NADPH was monitored at A_340 nm_.

### Insulin Disulfide Reductase Assay

The insulin disulfide reductase assay was used to measure the reduction of insulin disulfides by dithiothreitol (DTT) in the presence of Trx-1 or CLIC1 following the method described in [43]. The reaction was performed in 50mM Tris, 2 mM EDTA buffer (pH 7.5) containing 0.13 mM insulin, 0.33 mM DTT and 5 uM of Trx-1 or CLIC1. The change in solution turbidity due to insulin reduction was measured by monitoring absorbance at Lambda_650 nm_ over a period of 30 minutes.

### Glutathione-S-Transferase enzymatic activity of CLIC1

The enzymatic activity of CLIC1 monomeric (WT) protein in the presence of 1-chloro-2,4-dinitrobenzene (CDNB); p-nitrophenyl acetate and *trans*-octenal were performed following protocols described in [44]–[46]. Briefly, CLIC1 protein (5 uM final concentration) was added to a mixture containing 0.1 M potassium phosphate buffer (pH 6.5), 1 mM substrate (CDNB or p- nitrophenyl acetate or *trans*-octenal). The reaction was initiated by addition of 1 mM GSH. The GS-substrate conjugate was measured at A_340 nm_.

### Glutaredoxin-like Activity of CLIC1 using Sodium Selenite

The assay was performed following the method in [47]. Briefly, CLIC1 or Grx-1 (5 uM final concentration) was added to a mixture of 0.1 mM Tris-HCl buffer (pH 7.5) containing 1 mM EDTA, 200 uM NADPH, 50 nM GR, 0.1 mg/mL bovine serum albumin and 15 uM sodium selenite. The mixture was incubated for 5 minutes at 37°C, with the reaction initiated by addition of 50 uM GSH. Consumption of NADPH was monitored at A_340 nm_.

### Assay for Dehydroascorbic Acid Reductase (DHAR) Activity of CLIC1

The assay was performed following method in [48]. Briefly, CLIC1, CLIC4 or HcTrx-5 (5 uM final concentration) was added to 137 mM sodium phosphate buffer (pH 7.5), containing 0.35 mM NADPH, 50 nM GR and 2 mM GSH. The mixture was incubated for 1 minute at 30°C prior to initiation of reaction with 1 mM DHA. Consumption of NADPH was monitored at A_340 nm_.

### Ion Channel Blocker Drug and Cholesterol Experiments

CLIC1 (5 uM final concentration) was incubated with 10 uM IAA-94, A9C, DIDs or saxitoxin for 1 hour prior to performing the HEDS enzyme assay as outlined above. Similarly, 5 uM of CLIC1 in 156 uL of 5 mM potassium phosphate buffer (pH 7.5) was incubated with 0.4, 0.8 and 1.6 mM of cholesterol (34 mM cholesterol dissolved in ethanol) for 1 hour on ice (as previously described in [11]) prior to use of the protein sample in the HEDS assay.

## Results

### CLIC proteins show glutathione-dependent oxidoreductase activity

**Figure 2:**
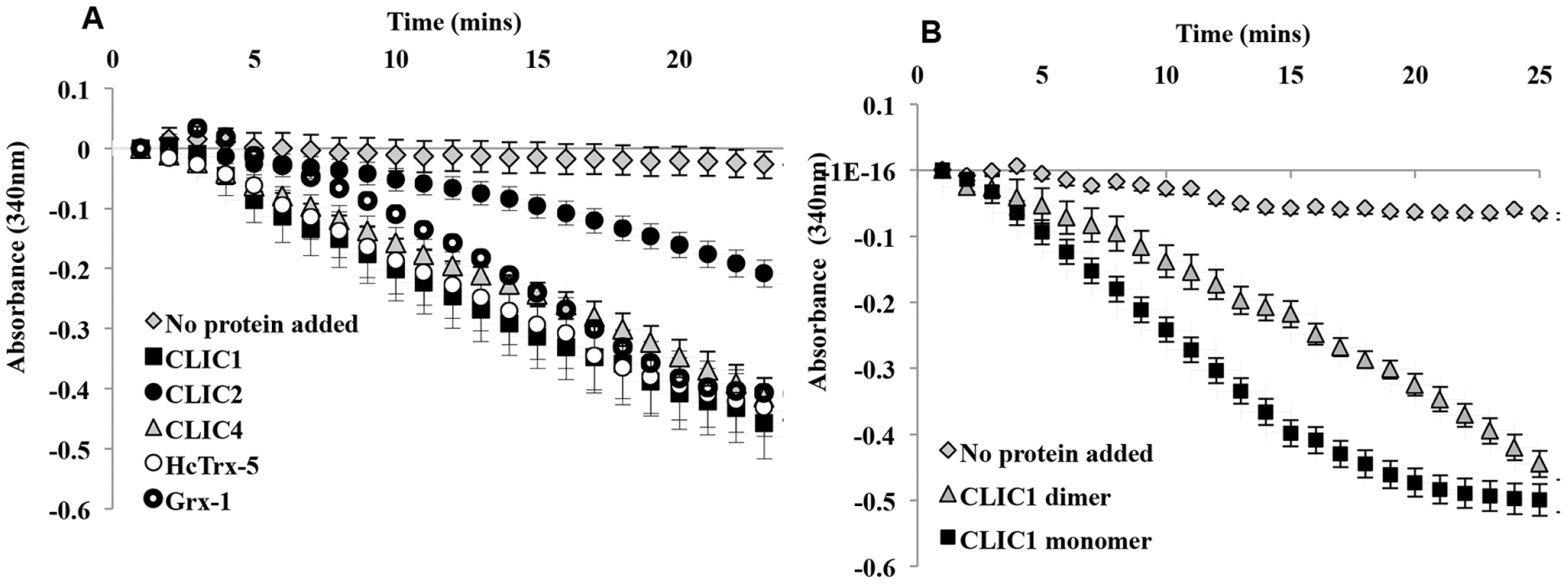
Oxidoreductase activity of the CLIC proteins. Oxidoreductase enzymatic activity was measured using 5 uM of CLIC proteins or HcTrx-5 or Grx-1, 250 uM NADPH, 1 mM HEDS and 50 nM GR. The reaction was initiated by the addition of 1 mM GSH and the absorbance of NADPH was monitored at A_340 nm_. Reaction conditions: 5 mM potassium phosphate with 1 mM EDTA, pH 7, at 37°C. (**A**) Activity of CLIC1, CLIC2 and CLIC4 compared to HcTrx-5 and Grx-1 (positive controls). (**B**) Activity of 5 uM CLIC1 dimer compared to 5 uM CLIC1 monomer. *Error bars* represent the S.E. of at least three independent measurements.

HEDS, 2-hydroxyethyl disulphide, is a low molecular weight compound found to act as a specific and sensitive substrate, suitable for use in assaying glutaredoxin enzymatic activity [35], [36], [49]. The HEDS assay was therefore employed in the current study to test for similar enzymatic activity by members of the CLIC protein family. As seen in Fig. 2A, consumption of NADPH increases (resulting in a decreased A_340 nm_) in the presence of the positive controls HcTrx-5 and Grx-1, well-known glutathione-dependent oxidoreductases. Similar consumption of NADPH is observed when CLIC1, CLIC4 and to a lesser extent CLIC2, are substituted for HcTrx-5 in the HEDS assay. This indicates that all three proteins reduced the HEDS substrate when coupled with glutathione (GSH) and glutathione reductase (GR) in the presence of NADPH. However CLIC2 is less active than CLIC1 and CLIC4.

Upon oxidation, soluble CLIC1 forms a non-covalent dimer, where the N-terminal thioredoxin fold domain structure is completely altered, disrupting the glutaredoxin-like active site [13]. The dimer is stabilized via an intramolecular disulfide bond between Cys24 and Cys59. The Cys59 residue is unique to CLIC1 and corresponds to a conserved alanine residue in the other five CLIC proteins [13]. The dimer form of CLIC1 was therefore tested for oxidoreductase enzymatic activity in the HEDS assay system. The CLIC1 dimer was found to reduce the HEDS substrate and demonstrated a similar rate of oxidised NADPH production of 0.02 uM/min, compared to monomeric CLIC1, with a rate of 0.03 uM/min (Fig. 2B).

Substitution of the HEDS substrate with the following three common GST substrates CDNB, p-nitrophenyl acetate or *trans*-octenal, in the enzyme assay system, with CLIC1 wild type protein did not result in any detectable enzymatic activity (data not shown).

### Oxidoreductase activity of CLIC proteins is glutathione-dependent

**Figure 3:**
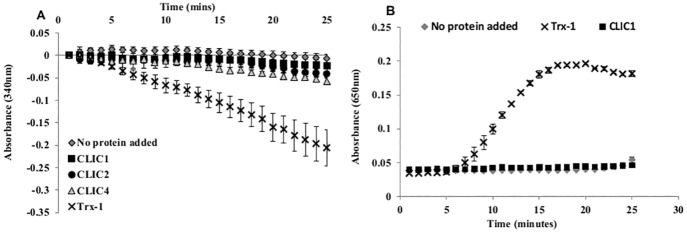
Glutathione-dependant activity of the CLIC proteins. (**A**) The reaction mixture contained 2 mM EDTA in 0.1 M Tris-HCl (pH 7.5), 5 uM reduced CLIC1, CLIC2 or CLIC4 (WT) protein, 200 uM NADPH, 750 uM HEDS, 50 nM TrxR and 5 uM Trx-1 (included as a positive control). (**B**) Insulin disulfide reductase assay to determine catalytic activity of Trx-1 and CLIC1 based on solution turbidity monitored by A_650 nm_ over 30 minutes.

Thioredoxins were the first antioxidants identified in cells, and are known to act as general protein disulfide reductase enzymes [50]–[52]. Thioredoxins are generally maintained in a reduced state in cells by accepting protons from NADPH via the enzyme thioredoxin reductase (TrxR) [35], [36], [52]. In order to determine whether CLIC protein enzymatic activity is linked to the TrxR system, CLIC1, CLIC2 and CLIC4 were assayed in a system containing TrxR, in place of GR. As expected, thioredoxin-1 (Trx-1) reduced the HEDS substrate when coupled with TrxR, evidenced by a reduction in NADPH absorbance over time, as seen in Fig. 3A. However CLIC1, CLIC2 and CLIC4 were unable to reduce the HEDS substrate in the presence of TrxR, demonstrating the CLIC proteins are not substrates for the thioredoxin system and hence cannot regain their reduced state.

Another common assay used to assess oxidoreductase activity by the thioredoxins is the insulin disulfide reductase assay as described by Holmgren (1979) [43]. In this assay the reduction of insulin disulfides by DTT is catalysed by Trx-1, resulting in increased solution turbidity via precipitation of the free insulin B chain [43]. CLIC1 was found to have no catalytic activity in this system when compared to Trx-1 (Fig. 3B).

### Conserved Cys24 is essential for CLIC1 oxidoreductase activity

**Figure 4:**
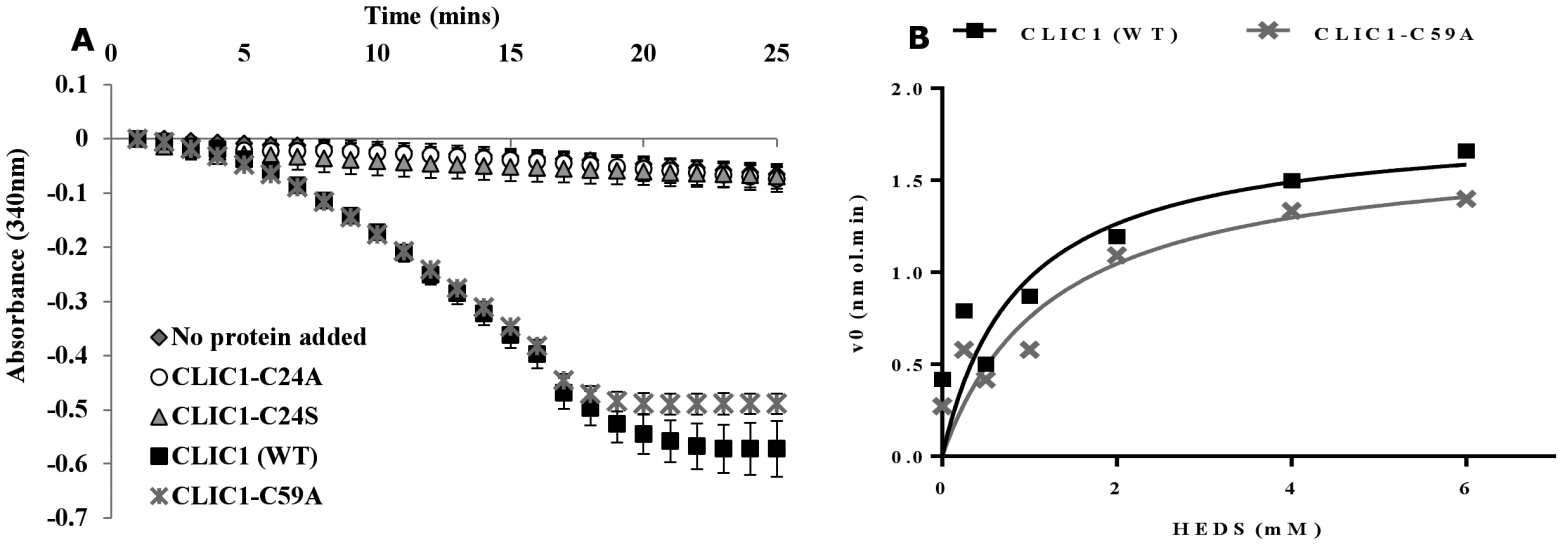
Comparison of the oxidoreductase activity of CLIC1 (WT) monomer and CLIC1-Cys mutants. (**A**) The reaction contained 1 mM EDTA in 5 mM potassium phosphate (pH 7), 250 uM NADPH, 50 nM GR, 1 mM HEDS and 5 uM of reduced CLIC1 (WT), CLIC1-C24A, CLIC1-C24S or CLIC1-C59A. The mixture was incubated for 5 mins at 37°C before initiation of the reaction with the addition of 1 mM GSH followed by monitoring NADPH absorbance at A_340 nm_. *Error bars* represent the S.E. of at least three experimental repeats. (**B**) A reaction of 5 uM of CLIC1 (WT) reduced monomer or CLIC1-C59A protein, 250 uM NADPH, HEDS (0, 0.25, 0.5, 1, 2, 4 or 6 mM) and 50 nM GR. The reaction was initiated by the addition of 1 mM GSH and the absorbance of NADPH was monitored at A_340 nm_. The reaction conditions where 5 mM potassium phosphate with 1 mM EDTA, pH 7, at 37°C.

In CLIC1; Cys24 represents the monothiol residue within the enzyme active site. In order to confirm Cys24 is the key active cysteine residue involved in CLIC1 oxidoreductase activity, mutant versions of CLIC1 were assayed, with Cys24 mutated to either alanine (C24A) or serine (C24S). In addition, Cys59, which forms an intramolecular disulphide bond with Cys24 in the CLIC1 dimer, was also mutated to alanine (C59A), and tested in the HEDS assay. Both Cys24 mutants of CLIC1, C24A and C24S were found to have no enzymatic activity in the HEDS assay (Fig. 4A). However the mutant C59A was capable of reducing the HEDS substrate in the presence of GR with a Km of 1.25±0.65 uM, which is indistinguishable to that of the wild type CLIC1 monomer (Km of 1.28±0.65 uM) (Fig. 4B).

### Sodium Selenite and Dehydroascorbic acid as substrates for CLIC1

**Figure 5:**
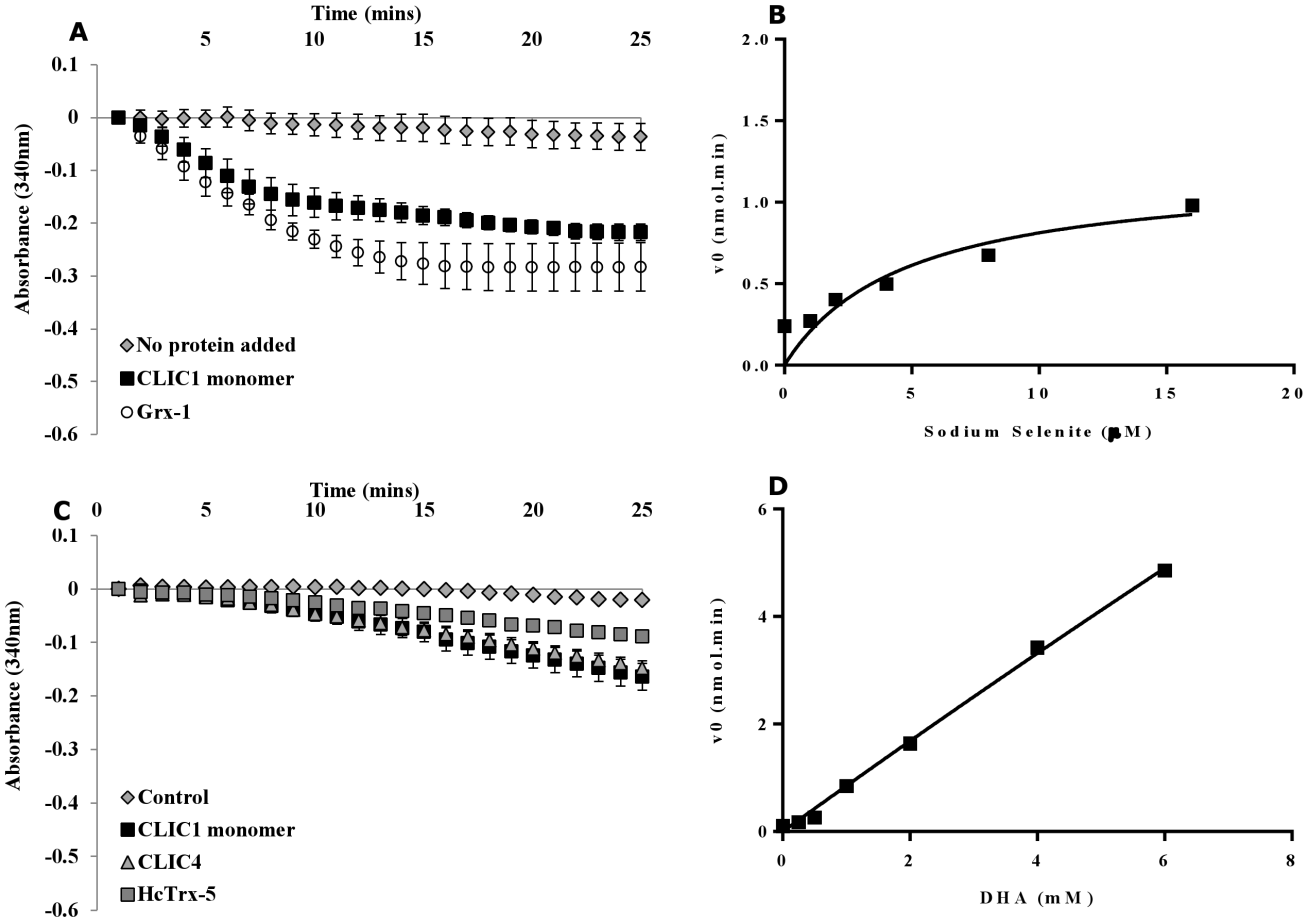
Sodium selenite and dehydroascorbic acid as substrates for CLIC1. (**A**) The oxidoreductase enzymatic reaction using sodium selenite as a substrate was performed in 0.1 mM Tris-HCl (pH 7.5) with 1 mM EDTA containing 200 uM NADPH, 50 nM GR,15 uM sodium selenite, 0.1 mg/mL BSA and 5 uM CLIC1(WT) reduced monomer or 5 uM Grx-1 as a control. The reaction was initiated by the addition of 50 uM GSH at 20°C with consumption of NADPH measured at A_340 nm_. *Error bars* represent the S.E. of at least three experimental repeats. (**B**) The reaction was performed in 0.1 mM Tris-HCl (pH 7.5) with 1 mM EDTA containing 200 uM NADPH, 50 nM GR, 5 uM CLIC1 (WT) reduced monomer and sodium selenite (0, 1, 2, 4, 8 or 16 uM). The initiation of the reaction was achieved by adding 50 uM GSH at 20°C where the consumption of NADPH was measured at A_340 nm_. (**C**) The oxidoreductase enzymatic reaction using DHAR as a substrate was performed in 137 mM sodium phosphate buffer (pH 7.5) containing 2 mM EDTA, 0.35 mM NADPH, 50 nM GR, 2 mM GSH and 1 mM DHA. The reaction was initiated after addition of 5 uM reduced CLIC1, CLIC4 or HcTrx-5 (as control). Consumption of NADPH was measured at A_340 nm_. *Error bars* represent the S.E. of at least three experimental repeats. (**D**) DHAR activity of the CLIC proteins was determined using 137 mM sodium phosphate buffer (pH 7.5) with 2 mM EDTA, 0.35 mM NADPH, 50 nM GR, 2 mM GSH and DHA (0, 0.25, 0.5,1, 2, 4 or 6 uM). The reaction was initiated after the addition of 5 uM CLIC1 (WT) protein and the NADPH consumption was monitored at A_340 nm_.

Glutaredoxins are known to act on a number of substrates such as selenium compounds [47] as well as dehydroascorbic acid (DHA) [35], [36], [48]. In order to investigate the ability of CLIC1 protein to reduce the selenite anion, a glutaredoxin-like activity assay was carried out in the presence of human Grx-1 or CLIC1 (WT) and sodium selenite (Na_2_SeO_3_) as the substrate, with the reaction initiated by the addition of GSH. In the presence of CLIC1 or Grx-1, the consumption of NADPH is stoichiometric to the selenite anion, which suggests that CLIC1 was also able to reduce sodium selenite, in a manner similar to Grx-1 (Fig. 5A). Titration of the sodium selenite substrate between (0–16 uM) demonstrated that CLIC1 has a relatively high Km (4.81±3.00 uM) (refer to Fig. 5B), compared to the normal concentration of selenium found in most cells (<1 uM) [53]. This would suggest that the binding affinity of CLIC1 to sodium selenite is low and as a result, product formation is dependent on the availability of sodium selenite.

Glutaredoxins are known to be involved in the reduction of DHA to ascorbate, which is a vital process for normal cellular function [35], [36]. We investigated the ability of the CLIC proteins to catalyse the reaction between GSH and DHA. In Fig. 5C, it can be seen that NADPH consumption increased in the presence of CLIC1 or CLIC4 and demonstrated similar activity to *HcTrx-5,* a known dehydroascorbate reductase (DHAR) from the parasitic worm *Haemonchus contortus* [41]. Kinetic studies using 5 uM CLIC1 protein and different concentrations of DHA (0-6 uM) indicate a linear relationship (Fig. 5D). This indicates CLIC1 has a strong binding affinity for DHA, suggesting soluble CLIC1 would be saturated by DHA under normal intracellular conditions.

### Inhibition of CLIC1 enzymatic activity by chloride ion channel blocker drugs but not cholesterol

**Figure 6:**
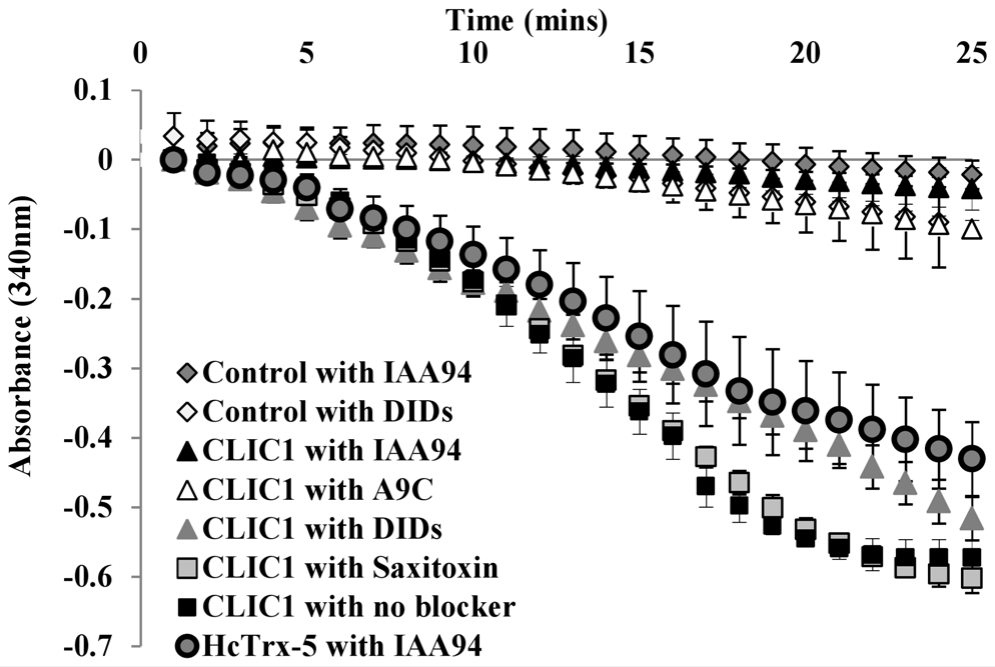
Effect of chloride ion channel inhibitor drugs on the oxidoreductase enzymatic activity of CLIC1. 5 uM of CLIC1 reduced (WT) or HcTrx-5 protein was incubated with 10 uM IAA-94, A9C, DIDS or Saxitoxin for ∼1 hour prior use of the protein in the assay. The enzyme assay mixture contained 250 uM NADPH, 1 mM HEDS, 50 nM GR in 5 mM potassium phosphate buffer with 1 mM EDTA, pH 7, at 37°C. The consumption of NADPH was monitored at A_340 nm_ post addition of 1 mM GSH. *Error bars* shown represent the S.E. of at least three experimental measurements.

IAA-94, A9C and DIDS are known chloride ion channel blockers. Electrophysiological studies have shown that both IAA-94 and A9C block CLIC1 ion channel activity in cells, while DIDS had no effect [22]. *In vitro* studies confirm that IAA-94 inhibits CLIC channels produced by adding recombinant soluble CLIC1 to artificial bilayers [8], [9], [51]. As seen in Fig. 6, both IAA-94 and A9C completely blocked the enzymatic activity of CLIC1, while DIDS had no effect. In addition, a known sodium ion channel blocker, saxitoxin, was found to have no effect on the enzymatic activity of CLIC1 in the HEDS enzyme assay.

**Figure 7:**
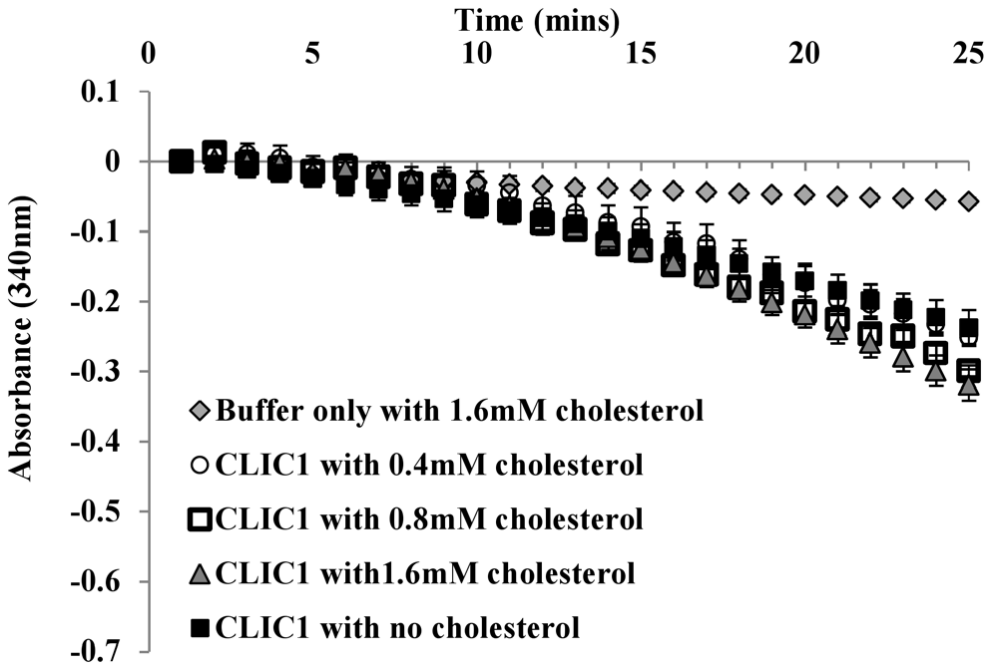
Effect of cholesterol on the enzymatic activity of CLIC1. 5 uM of CLIC1 monomer (WT) protein was incubated with 0.4, 0.8 and 1.6 mM cholesterol for ∼1 hour prior to its addition to a reaction mixture of 250 uM NADPH, 1 mM HEDS, 50 nM GR in 5 mM potassium phosphate buffer with 1 mM EDTA, pH 7, at 37°C. The consumption of NADPH was monitored at A_340 nm_ post addition of 1 mM GSH. Control included all the reaction components including 1.6 mM cholesterol, except with no CLIC1 protein. The *error bars* shown represent the S.E. of at least three experimental measurements.

We have recently shown that cholesterol is critical for the insertion and conductance of CLIC1 in artificial membrane systems and that pre-incubation of CLIC1 with 0.4, 0.8 or 1.6 mM cholesterol prior to addition of the protein to membranes inhibited CLIC1 membrane insertion and ion conductance [11]. We therefore assessed whether cholesterol could also regulate CLIC1 enzymatic activity. Pre-incubation of the protein with cholesterol resulted in no change in CLIC1 enzymatic activity in the HEDS enzyme assay (refer to Fig. 7).

## Discussion

Members of the CLIC family are soluble proteins capable of spontaneously inserting into lipid membranes - in particular intracellular membranes - to form chloride selective ion channels [8], [14], [20], [54]. To date, characterization of these proteins has focused on their membrane insertion and their ion channel activity, with no distinct function assigned to their soluble form.

In the current study we have shown that CLIC proteins can act as glutathione-dependent oxidoreductases in the HEDS enzyme assay. This assay system is considered a characteristic assay for the glutaredoxin proteins which act as enzymes by deglutathionylating the mixed disulphide between glutathione (GSH) and the beta-mercaptoethanol region of the HEDS reagent [55]. This *in vitro* demonstration of Grx-like activity for CLICs 1, 2 and 4 suggests the soluble form of these proteins may act to catalyse the reduction of disulfides and thus function as oxidoreductases in cells (Fig. 2A). The glutaredoxin-like activity of CLIC1 was further supported by its lack of activity in the common Trx disulfide reductase assay (Fig 3B), as well as, its lack of demonstrable enzymatic activity against the common GST substrates, CDNB, p- nitrophenyl acetate and *trans*-octenal.

Several dithiol glutaredoxins (e.g. human Grx2) have been found to perform diverse activities, including being reduced by thioredoxin reductase (TrxR) as well as GSH and glutathione reductase (GrxR) [56]. However CLICs 1, 2 and 4 were not reduced by the selenoenzyme, thioredoxin reductase (TrxR) (Fig. 3A). Of particular note was CLIC2, which as a dithiol protein containing the additional cysteine residue in its enzyme active site, may be expected to show similar activity to these dithiol Grxs. Given the lack of activity by all three CLIC proteins, one could speculate that they have GSH-dependent enzymatic activity that is distinct to the dithiol glutaredoxins, however further comparative studies are needed in order to ascertain a distinct CLIC enzymatic profile.

Of the three CLIC proteins studied, CLIC2 demonstrated the lowest level of enzymatic activity in the HEDS assay, compared to either CLIC1 or CLIC4. We note that a previous study found no significant enzymatic activity for CLIC2 in the HEDS enzyme assay [57]. The apparent lower activity of CLIC2 compared to CLIC1 and CLIC4 may be related to the variation in the active site between these proteins, with CLIC2 containing the dithiol motif (CPFC), whilst both CLIC1 and CLIC4 contain the monothiol motif (CPFS). However, further studies are needed in order to establish a distinct dithiol catalytic mechanism by CLIC2 compared to the monothiol members CLIC1 and CLIC4.

Enzymes exhibiting a glutaredoxin-like activity require an active site cysteine. In the glutaredoxin system, the first cysteine residue in the G-site motif is reported to attack the sulfur atom in disulfide bridges - as occurs within glutathione mixed disulfide bonds - and therefore promotes thiol transfer [58]. Similarly the GST-omega and -beta groups demonstrate glutaredoxin-like activity in the HEDS enzyme assay with the first cysteine residue in their G-site also found to be essential for their enzymatic activity [59], [60]. Our mutagenesis results show that Cys24 is the essential catalytic cysteine, as expected from the CLIC1 structure [8]. Although Cys59 is essential for the transition of CLIC1 from the reduced monomer to the oxidized dimer state [13], mutation of Cys59 to alanine does not alter the enzymatic activity of the soluble monomer. This confirms Cys24 in CLIC1 as the central redox catalytic residue, essential for the enzymatic function of CLIC1, with no apparent involvement of Cys59. We note that mutation of either Cys24 or Cys59 to serine reduced the ability of CLIC1 to form ion channels in artificial bilayers [13]. Thus the cysteine residues that are essential for the enzymatic activity of the soluble form of CLIC1 are a subset of the cysteines that are necessary for its ion channel activity.

The glutathione-dependent oxidoreductase activity of the oxidised CLIC1 dimer is an unexpected finding (Fig. 2B). The structure of this soluble form of CLIC1 is radically different from the reduced CLIC1 monomer [13]. The N-terminal domain of the dimer no longer resembles glutaredoxin and the reactive cysteine, Cys24, forms a disulphide bond with Cys59. Thus, this dimeric form of CLIC1 should not possess the same catalytic activity as the reduced monomer. The solution of this conundrum comes by examining the assay conditions, which include 1 mM GSH. These reducing conditions will rapidly convert the CLIC1 oxidised dimer back into the reduced monomeric form as the structural transition is fully reversible [13]. Thus, over the thirty minute timeframe of the experiment the enzymatically active molecule will be the regenerated CLIC1 monomer. For clarity, we note that the oxidised CLIC1 dimer does not have a GST fold and certainly does not resemble the GST dimer, which is the normal form for all GSTs including GST-omega class [33].

Phylogenetic studies have shown that the plant dehydroascorbate reductases (DHAR) are the closest relatives of the CLIC protein family [61]. Thus, these plant DHAR proteins are predicted to adopt a three-dimensional structure similar to the soluble form of CLIC1 [61], [62]. A recent study has demonstrated oxidoreductase activity in the DHAR from *Populus tomentosa* [61]. Upon alanine substitution of the Cys20 residue which is located in the predicted GSH binding site in the protein PtoDHAR2, its reductase activity was abolished [61]. These findings correlate closely with our results for the two CLIC1 mutants (C24A and C24S) (Fig. 3), where both were inactive in the HEDS enzyme assay, while the C59A mutant remained active. We also note that a DHAR from *Arabidopsis thaliana*, AtDHAR1, has been cloned and transiently expressed in mammalian cells where it shows CLIC-like ion channel activity [62].

In cells, selenide, which is the reduced form of selenium, undergoes redox cycling with oxygen and thiol causing a significant production of reactive oxygen species (ROS) [51]. In turn, increased generation of ROS and superoxides leads to cellular damage and can induce apoptosis. According to our findings (Fig. 4A), CLIC1 is able to metabolise sodium selenite in a manner similar to Grx-1. This finding supports the hypothesis that soluble CLIC1 may function as an antioxidant and an oxidoreductase enzyme in cells. Ascorbic acid or vitamin C is found in high concentration in some body tissues and is believed to be an effective scavenger of superoxide, hydroxyl radical and hydrogen peroxide [47]. The metal-catalyzed oxidation products of ascorbate are DHA and H_2_O_2_, which are highly toxic to cells and have been linked to many diseases including senile cataracts in ocular lenses [63]. Glutaredoxins, being redox active proteins, demonstrate DHAR activity by catalyzing the reactions between GSH and DHA and thus reduce the DHA back to ascorbate. CLIC1 demonstrated the same oxidoreductase activity by reducing DHA (Fig. 5B). From these results, one could speculate that members of the CLIC family serve a protective function in cells by metabolizing substrates such as sodium selenite and DHA and thus maintaining the intracellular levels of ascorbate. Given the low binding affinity of CLIC1 for sodium selenite, DHA is the more likely physiological substrate for the CLIC proteins. DHAR activity of the CLIC proteins is consistent with their close evolutionary relationship with the plant DHAR proteins [14], [61], [62]. If this putative activity by the CLICs is considered within the context of the ocular lens, reduction of DHA by CLIC proteins could aid in preventing selenite cataract formation.

An intriguing finding of our work was the inhibitory effect on CLIC1's enzymatic activity in the HEDS enzyme assay by the chloride ion channel blockers IAA-94, A9C but not DIDS. These findings are consistent with the structural and evolutionary relationship between the GST and CLIC families as IAA-94 is a homologue of ethacrynic acid [64] which is a known inhibitor of the enzymatic activity of a number of GSTs [65] a point also noted on the determination of the structure of CLIC1[8].

The inhibition of CLIC1 enzymatic activity coincides with previous electrophysiological experiments that demonstrated CLIC1 channel activity was blocked by IAA-94 and A9C but not by DIDS [22]. CHOK1 cells grown in the presence of IAA-94 and A9C resulted in their arrest at G2M phase of the cell cycle, but this was not the case for DIDS. Given that these drugs are membrane permeable, their cellular inhibitory effects and arrest of the cell cycle progression, could be due to inhibition of CLIC1 enzymatic activity rather than directly blocking the integral membrane form of the CLIC1 channel.

This concurrence of enzymatic inhibitory profile and channel function blockage has profound consequences. The structural transition of CLIC1 from the soluble form to the integral membrane form is likely to result in a complete disruption of the thioredoxin-like N-terminal domain of the CLIC1 GST fold [8], [12], [13], [42], [54]. Thus, if IAA-94 binds to the soluble form of CLIC1 in the cleft between the N-domain and the C-domain, as seen in the structures of GST proteins [66], [67] then it is unlikely to bind directly to the integral membrane form as this binding site is unlikely to exist. This leaves two possible explanations for the inhibition of the CLIC1 ion channel by IAA-94: (1) the inhibitor binds to a new, distinct site on the integral membrane form of CLIC1 or (2) the inhibition of the channel is mediated by the inhibition of the enzymatic activity of the soluble form of CLIC1. Binding of the inhibitors to a new, distinct site seems unlikely, albeit possible. The more likely explanation is that the inhibitors, IAA-94 and A9C, act by binding near the active site of the soluble form of CLIC1 thus inhibiting its enzymatic activity and consequently its channel activity.

How can the soluble CLIC1 enzyme control the ion channel function of the membrane-inserted form of CLIC? *In vitro* experiments have shown that CLIC1 (and other CLIC proteins) alone can form electrophysiologically active anion channels in artificial bilayers where the electrophysiological properties resemble those of the CLIC currents observed in cells [9], [10], [15]. Thus, *in vitro*, the CLIC protein must auto insert into the bilayer to form the channel. It is possible that the CLIC1 enzymatic activity can either control this membrane insertion process or that once some CLIC1 has inserted and formed a channel, then the remaining soluble CLIC1 controls the channel via its enzymatic activity. In cells, it is also possible that the soluble CLIC enzyme controls other channels as has been shown for CLIC2 and the ryanodyne receptor Ca^2+^ release channel [57]. We note that it is still possible that the inhibitors, IAA-94 and A9C, bind directly to the channel form of CLIC1, however, this site would be different from the one observed in the soluble form, as noted above.

Glutathionlyation is a reversible modification of proteins in which a mixed disulfide bond forms between glutathione (GSH) and a cysteine residue of a protein. It is considered a critical process for signal transduction as well as cellular homeostasis, where it plays an essential role in protecting cysteine residues from oxidative damage [68], [69]. Glutaredoxins and GST-omega-1 were found to catalyse protein deglutathionylation in order to maintain cellular sulfhydryl homeostasis [59], [69]. Recent studies suggest that the change in glutaredoxin levels affect protein glutathionylation status and, subsequently, downstream signalling events [61].

Given these activities by the Grxs, we could also expect that members of the CLIC family are capable of carrying out target protein de/glutathionylation activity. This is supported by the X-ray crystallographic studies that reveal an open slot adjacent to the GSH binding site in CLIC1 that is large enough to accommodate a protein substrate [23]. De/glutathionylation may well be the mechanism by which CLIC proteins control ion channel activity and other cellular processes [14], [70].

In conclusion, members of the CLIC protein family, which are known to function as ion channels when integrated into membranes, also demonstrate monothiol glutaredoxin-like enzymatic activity when in their soluble form. This supports an additional role for these proteins in the cellular processes of detoxification and oxidoreduction. Furthermore, the enzymatic activity of CLIC1, appears to be distinct to its ion channel activity, as demonstrated by cholesterol's regulation of the latter activity but not the former, which would support classification of the CLICs as moonlighting proteins [38]. Finally, the fact that the same CLIC1 channel blockers inhibit CLIC1 enzymatic function suggests that the enzymatic properties of CLIC1 may also control the function of the channel form.
